# Development of diabetes-specific quality of life module to be in conjunction with the World Health Organization quality of life scale brief version (WHOQOL-BREF)

**DOI:** 10.1186/s12955-017-0744-3

**Published:** 2017-08-23

**Authors:** Chung-Ying Lin, Tsung-Ying Lee, Zih-Jie Sun, Yi-Ching Yang, Jin-Shang Wu, Huang-Tz Ou

**Affiliations:** 1 Department of Rehabilitation Sciences, Faculty of Health and Social Sciences, Hong Kong Polytechnic University, Hung Hom, Hong Kong; 2 Institute of Clinical Pharmacy and Pharmaceutical Sciences, College of Medicine, National Cheng Kung University, Tainan, Taiwan; 3 Department of Family Medicine, National Cheng Kung University Hospital, Tainan, Taiwan; 4 Department of Family Medicine, National Cheng Kung University Hospital, Dou-Liou Branch, Dou-Liou, Taiwan; 5 Department of Family Medicine, College of Medicine, National Cheng Kung University, Tainan, Taiwan; 6 Department of Pharmacy, College of Medicine, National Cheng Kung University, Tainan, Taiwan; 7 Department of Pharmacy, National Cheng Kung University Hosiptial , Tainan, Taiwan

**Keywords:** Diabetes, Health-related quality of life, WHOQOL-BREF, Reliability, Validity, Chinese

## Abstract

**Background:**

Although numerous health-related quality of life (HRQoL) instruments are available for patients with diabetes, the length of these measures may limit their feasibility to routine practice. Also, these measures do not distinguish items for generic and diabetes-specific HRQoL. This study was aimed to develop a diabetes-specific quality of life questionnaire module (DMQoL) to be in conjunction with the World Health Organization Quality of Life scale brief version (WHOQOL-BREF).

**Methods:**

One hundred seventeen patients with diabetes were enrolled from a medical center in Taiwan. The item content of DMQoL was constructed based on an extensive review of existing HRQoL instruments for diabetes, expert discussions and patient interviews. A series of psychometric tests were conducted to ensure the reliability and validity of DMQoL. The WHOQOL-BREF served as an existing HRQoL measure for construct validity testing. The response scale of DMQoL was adopted from the 5-point Likert scale of WHOQOL-BREF.

**Results:**

A total of 10 items without ceiling or floor effects were selected from 20 items. Exploratory factor analysis (EFA) with parallel analysis and Rasch analysis concluded that the 10 items were embedded in the same underlying concept. The corrected item-total correlations and factor loadings from EFA were all above 0.4. The internal consistency of the 10 items was satisfactory (Cronbach’s α = 0.84). The DMQoL total score was moderately correlated with that of WHOQOL-BREF (*r* = 0.48, *p* < 0.001). The known-group validity showed that patients with HbA1c ≤ 7% had significantly higher mean scores of DMQoL than did those with HbA1c > 8% (3.66 ± 0.47 vs. 3.41 ± 0.53; *p* = 0.037).

**Conclusions:**

The DMQoL with only 10 items is developed and it is sensitive to the change of diabetes progression in early phases (e.g., glycemic changes). The combination of WHOQOL-BREF and DMQoL provides a comprehensive picture of overall HRQoL in patients with diabetes and enhance the instrument’s ability to detect clinically meaningful changes in diabetes.

**Electronic supplementary material:**

The online version of this article (doi:10.1186/s12955-017-0744-3) contains supplementary material, which is available to authorized users.

## Background

The number of people living with diabetes globally is projected to increase from about 170 million in 2000 to 370 million in 2030 [1]. Diabetes is a progressive and demanding disease with serious short- and long-term consequences. Acute episodes of hyperglycemia (e.g., diabetic ketoacidosis) and hypoglycemia, fear of hypoglycemia, change in lifestyle, and fear of long-term microvascular complications (e.g., retinopathy, nephropathy) and macrovascular complications (e.g., cardiovascular diseases; CVD) may diminish health-related quality of life (HRQoL) in such individuals [2]. As compared to individuals without diabetes, diabetes patients have lower HRQoL [3]. Diabetes can overwhelm patients because of its management, acute physical distress of hyperglycemic or hypoglycemic episodes, and chronic physical distress of diabetes-related complications. Hence, for chronic illnesses such as diabetes, assessing how the disease influences patients’ lives and the burden of the demands of the disease on patients is essential for planning care and establishing therapies.

HRQoL instruments subjectively or objectively measure different domains of health, which may include physical, mental and social health [4]. Improving HRQoL is an important health outcome for health interventions and therapies [5]. For chronic illnesses such as diabetes, HRQoL has become increasingly important in the assessment of disease severity, the evaluation of interventions, and the allocation of healthcare resources. Two main approaches with different purposes have been used to describe and measure HRQoL: generic and disease-specific instruments [6]. Generic HRQoL instruments capture a broader construct of health, and thus, they are useful for comparing HRQoL across different disease areas and population groups. Generic instruments such as the World Health Organization Quality of Life Questionnaire (WHOQOL)-100 [7] are commonly used when clinicians want to compare HRQoL across groups with different types of conditions/illnesses. In addition, its short version, WHOQOL-BREF [7], is popular because of its efficiency in measuring HRQoL. WHOQOL-BREF, which contains 26 items, was developed with 15 international field centers to obtain an assessment tool that is applicable cross-culturally. Researchers have used WHOQOL-BREF to assess HRQoL in patients with diabetes [8–10], including Taiwan population [11].

However, generic HRQoL measures may be less sensitive to special problems of a certain disease such as diabetes, as compared to disease-specific measures [6]. With this regards, the generic and disease-specific measures of HRQoL are recommended to use together [6]. By doing this, healthcare providers can thoroughly assess the health condition of a patient with diabetes; generic HRQoL instruments can capture the general health status, while the diabetes-specific instruments assess the specific impacts of diabetes on the patient’s health [6]. The dimensions covered by diabetes-specific instruments vary, but generally include physical function, psychological function, social role fulfillment, diabetes control, and treatment satisfaction [12]. Although the combination of generic and diabetes-specific HRQoL instruments can provide a whole picture of HRQoL of patients, for practical purpose, it would be useful to have separated generic and disease-specific instruments that can be used according to clinical needs. For example, a diabetes-specific instrument can be used stand-alone to understand the impact of diabetes in detail for patients with diabetes, while a generic measure can be used to assess general health in these patients.

Although numerous disease-specific HRQoL measures (Additional file 1: Appendix 1) exist for patients with diabetes, the length of these measures (e.g., up to 39–51 items [13–16]) may limit their feasibility to routine practice. The lengthy issue may be overcome by using some stand-alone, diabetes-specific HRQoL instruments with few items that can be used in routine clinical settings [17]. However, the following reasons suggest developing a new diabetes-specific HRQoL instrument. First, existing diabetes-specific questionnaires [13–16] have some domains overlapped with generic HRQoL instruments (e.g., WHOQOL-BREF). Second, developing a diabetes-specific questionnaire that functions in two ways is needed: a screening tool in surveys and a supplement for a generic instrument to assess thorough HRQoL information. Third, comparing a diabetes-specific HRQoL instrument with an existing generic instrument is needed to ensure the HRQoL construct [18, 19]. Fourth, no factor analysis has been done for those diabetes-specific HRQoL instruments with few items [17]. Against this background, the present study aims to develop a diabetes-specific HRQoL instrument that is stand-alone, allows quick and easily identification of the effects of diabetes on the HRQoL of patients in daily practice, and can be supplementary to WHOQOL-BREF for assessing overall HRQoL in such individuals.

## Methods

### Participants

Our target population was people with diabetes. Our participants were prospectively recruited from National Cheng Kung University Hospital (NCKUH) during September 2015 to August 2016. They met the following criteria: (1) aged over 20 years; (2) had been diagnosed with diabetes; (3) had the ability to understand spoken Mandarin or Taiwanese; (4) was an outpatient of the Department of Family Medicine, NCKUH. The exclusion criteria were: (1) had mental disorders; (2) suffered a major traumatic event, such as divorce, separation, or death of someone close, at least 6 months prior to data collection. This is because the people experiencing these life events are likely to have negative emotions (e.g., depression, sad, anxiety), which may have deleterious effects of their psychological well-being or HRQoL [20–22]. The Institutional Review Board of NCKUH approved this study before commencement (A-ER-103-298). All the participants voluntarily agreed to participate in the study with each provided written informed consent.

Each participant was assessed by the second and third authors who have been trained for evaluating HRQoL and clinical outcomes. Demographic information (e.g., age, education level) was obtained using an information sheet. Data on laboratory measures (e.g., low-density lipoprotein, HbA1c, estimated glomerular filtration rate; e-GFR), clinical diagnoses of diabetes-related complications, and HRQoL were collected at the same time for each patient. Diabetes-related complications, including cerebral and cardiovascular diseases (if patients had confirmed clinical diagnosis of stroke, myocardial infarction, coronary heart disease, or heart failure), peripheral vascular disease; (if patients had confirmed clinical diagnosis of foot ulcers, claudication, amputation, embolism of the lower extremity, or ankle-brachial index <0.9), retinopathy, nephropathy (if patients were at chronic kidney disease stage 3–5, had eGFR <60 or microalbuminuria confirmed by an albumin-to-creatinine ratio of 30 mg/g), and neuropathy (confirmed by a nerve conduction velocity test, or if patients had confirmed clinical diagnosis of neuropathy), were collected by reviewing the Electronic Medical Records of NCKUH.

### Development of target instrument: Diabetes mellitus-specific quality of life questionnaire, DMQoL

The development of DMQoL consisted of the following steps [23]: (1) a literature review and pooling of all possible items; (2) removal of redundant items; (3) setting up an expert panel to draft the first version; (4) sending the first version out for expert opinions; (5) setting up another expert panel to integrate the expert opinions to draft the second version; (6) use of the second version for a pilot test; (7) setting up a third expert panel to integrate the opinions of patients and practical issues identified in the pilot test; (8) development of the response scale.

For the first step, we identified 13 instruments (Additional file 1: Appendix 1) that measure HRQoL for people with diabetes, with a total of 467 items. Two researchers (i.e., the first and last authors) removed items with similar concepts and those not directly correlated with diabetes mellitus; a total of 66 items were retained. The expert panels in steps (3), (5), and (7) consisted of two pharmacists, two family doctors, and a psychometrician. The first expert panel condensed the 66 items to 22 items. The 22 items were used as the first draft of DMQoL and sent out for review to another external panel which consisted of 12 experts in diabetes (4 endocrinologists, 4 family doctors, 2 nurse educators, and 2 pharmacists) in Taiwan. Based on the comments from the experts, the second expert panel decided to drop two items, and made the second draft of DMQoL, containing 20 items (Additional file 1: Appendices 2 and 3). The second draft was sent out to 13 patients with different ages (6 aged over 65 years), educational levels (4 with college degree; 4 with high school degree; 4 with elementary school degree; 1 illiterate), and genders (7 males; 6 females) to review the second draft. Specifically, we asked the 13 patients to use a 4-point Likert scale to rate how well they understood the item descriptions. The pilot results showed that these item descriptions were understandable to the patients (mean score = 3.69 to 4). Based on the pilot results, the third expert panel decided that the 20 items were suitable for preliminary psychometric testing.

The response scale of DMQoL is the 5-point Likert scale of WHOQOL-BREF. That is, it uses the same descriptors designed for WHOQOL-BREF Taiwan version [24]. The DMQoL had a 5-point Likert scale to ensure consistency with the WHOQOL-BREF. This 5-point scale was also expected to capture nuanced changes in health that may be brought about by treatment better than a 3-point or 4-point scale [25].

### Instrument for concurrent validity: WHOQOL-BREF

WHOQOL-BREF Taiwan version with four domains (physical, psychological, social, and environment) was developed with cultural adaptation [26] according to the guidelines made by the World Health Organization [27]. Two items were added for the Taiwan version (one in the social domain and another in the environment domain), giving WHOQOL-BREF Taiwan version 28 items. Of the 28 items rated using a 5-point Likert scale, 26 are distributed in the four domains. The domain scores are calculated by using the following steps: (1) reverse the scores of items Q3, Q4, and Q26, (2) calculate the average score for the items in the same domain (e.g., physical domain contains Q3, Q4, Q10, and Q15 to Q18), and (3) multiply the average score by 4. Finally, we can get the domain scores in a 4–20 scale [26]. The two items not embedded in any domain are general questions: Q1 asks about overall HRQoL and Q2 asks about general health. Higher scores (including domain scores, Q1 and Q2 scores, and total scores) represent better HRQoL. Also, the internal consistency (α = 0.70 to 0.91) and the test-retest reliability (*r* = 0.76 to 0.80) of WHOQOL-BREF Taiwan version were acceptable [26]. The construct validity of WHOQOL-BREF Taiwan version was also supported (comparative fit index = 0.95) by confirmatory factor analysis [28].

### Analysis strategies

After all the items for DMQoL had been decided, we examined the following psychometric properties of DMQoL: (1) ceiling and floor effects; (2) construct validity; (3) internal consistency; (4) concurrent validity; (5) known-group validity.

For ceiling and floor effects, we reported the percentages of minimum (score of 1) and maximum (score of 5) ratings given by respondents. We omitted items whose ceiling/floor effect was larger than 20% [29], which suggests that they may not provide sufficient information. The construct validity was examined using two methods: exploratory factor analysis (EFA) combined with parallel analysis (PA) and Rasch analysis. We calculated the sample size based on the rule of thumb for the EFA; that is, 5–10 samples per item. Given that we aimed to develop less than 20 items, the acceptable sample size was between 100 and 200. Principal axis factoring was used as the extraction method in the EFA, and we compared the eigenvalues extracted from our data to those extracted from simulated samples (i.e., PA). A total of 200 samples were generated for the simulation. The number of extracted factors was decided based on how many eigenvalues extracted from our data were larger than those extracted from simulated samples [30, 31]. The rating scale model of Rasch analysis was used to analyze the item properties. Item fit was determined using information-weighted fit statistic mean square (infit MnSq) and outlier-sensitive fit statistic MnSq (outfit MnSq). A range of 0.5 to 1.5 suggests that an item is embedded in the latent construct (i.e., DMQoL), which is evidence of construct validity [28]. Also, corrected item-total correlation was calculated to determine whether each item was well connected with the entire concept; a value of >0.4 was considered acceptable.

For internal consistency, in addition to the traditional Cronbach’s α, Rasch analysis was used to obtain item separation and person separation reliability values. We anticipated that all the values for internal consistency would be larger than 0.7. For concurrent validity, WHOQOL-BREF was used as a criterion. Specifically, we tested the correlation between the score of DMQoL and the following scores in WHOQOL-BREF: Q1 and Q2, four domains, and total score. In addition, because age is a potential confounder of HRQoL [28], we additionally calculated the partial correlation that adjusted for age. Lastly, the known-group validity was tested using the laboratory data (e.g., HbA1c, cholesterol) and diabetes-related complications. For instance, we compared the DMQoL scores of patients with a better HbA1c (≤7%) with those of patients with a worse HbA1c (>8%). We hypothesized that patients with poorer glycemic control (using HbA1c as a marker of glycemia control) or diabetes-related complications would have lower HRQoL as compared to those who had achieved the glycemic target (i.e., HbA1c ≤ 7%) or without diabetes-related complications.

## Results

One hundred seventeen patients with diabetes (slightly more than half were males) were enrolled for the preliminary psychometric testing. Less than one fifth of them were aged 50 years or below (*n* = 21), and most of the patients controlled their diabetes quite well according to their HbA1c (32 were >8%), laboratory data, and data on the clinical diagnosis of diabetes-related complications (Table 1).

**Table 1:** Participant characteristics

Participant characteristics	Value
Age, years, median (IQR)	63 (55, 70)
Sex, male, n (%)	59 (50.4)
Education, ≥ College, n (%)	45 (38.5)
Diabetes complication^a^	
CCD, n (%)	16 (17.6)
PVD, n (%)	4 (4.4)
Retinopathy, n (%)	16 (17.6)
Nephropathy, n (%)	34 (37.4)
Neuropathy, n (%)	10 (11.0)
HbA1c, percentage, median (IQR)	7.50 (6.83, 8.30)
Cholesterol, mg/dl, median (IQR)^a^	159.50 (139.75, 183.00)
HDL, mg/dl, median (IQR)^a^	46.50 (36.00, 57.00)
LDL, mg/dl, median (IQR)^a^	99.00 (85.00, 121.00)
Triglyceride, mg/dl, median (IQR)^a^	122.00 (87.00, 162.25)
e-GFR, ml/min, median (IQR)^a^	90.00 (72.00, 90.00)

^a^with missing values

*Abbreviations*: *CCD* Cerebral and cardiovascular diseases, *PVD* Peripheral vascular diseases, *HDL* High-density lipoprotein-cholesterol, *LDL* Low-density lipoprotein-cholesterol, *TG* Triglyceride, *e-GRF* estimated Glomerular Filtration Rate

The frequencies of the responses to our designed items showed that 8 items had floor effects (i.e., > 20% of the respondents rated the item as 1) and 2 had ceiling effects (i.e., > 20% of the respondents rated the item as 5). Based on the distributions (Table 2), 10 items were retained for the following analyses, including EFA combined with PA, Rasch analysis, internal consistency, known-group validity, and concurrent validity (Table 3). EFA showed that there were two eigenvalues >1 (4.09 and 1.24) extracted from our data; however, PA showed that the mean eigenvalue using a 200-sample simulation was 1.50 for the first extraction and 1.34 for the second extraction. In addition, the simulated eigenvalues at the 95% confidence level were 1.62 and 1.43 for the first two extractions, respectively. Therefore, the second eigenvalue extracted from our data cannot be viewed as a separate factor, and thus we treated the 10 items as being embedded in one underlying concept. The subsequent Rasch analysis supported our results of EFA combined with PA: all the infit and outfit MnSq values were between 0.5 and 1.5. That is, each item fell within the same concept (Table 3). Furthermore, the corrected item-total correlation and factor loadings retrieved from the EFA were all above 0.4 (Table 3).

**Table 2:** Frequency of response on each item of DMQoL

Item #: Item description	Response; n (%)				
	1	2	3	4	5
1: Overall, how much does diabetes control influence your life?	25 (21.4%)	39 (33.3%)	19 (16.2%)	21 (17.9%)	13 (11.1%)
2: How satisfied are you with the time you spend on diabetes care?	1 (0.9%)	16 (13.7%)	28 (23.9%)	66 (56.4%)	6 (5.1%)
3 ^a^ : How satisfied are you with your expenses on diabetes care?	2 (1.7%)	5 (4.3%)	34 (29.1%)	67 (57.3%)	8 (6.8%)
4 ^a^ : How satisfied are you with the results of glycemic control?	2 (1.7%)	36 (30.5%)	33 (28.0%)	40 (33.9%)	4 (3.4%)
5 ^a^ : How satisfied are you with the results of the control of diabetes-related complications?	0 (0.0%)	8 (6.8%)	29 (24.8%)	63 (53.8%)	14 (12.0%)
6^a^: How much difficulty have you had in self-care in the management of diabetes?	51 (43.6%)	27 (23.1%)	13 (11.1%)	9 (7.7%)	16 (13.7%)
7 ^a^ : How satisfied are you with your diet control?	0 (0.0%)	10 (8.5%)	38 (32.5%)	62 (53.0%)	5 (4.3%)
8 ^a^ : How satisfied are you with your physical activities?	4 (3.4%)	28 (23.9%)	21 (17.9%)	56 (47.9%)	7 (6.0%)
9 ^a^ : How satisfied are you with your weight control?	3 (2.6%)	22 (18.8%)	30 (25.6%)	58 (49.6%)	3 (2.6%)
10 ^a^ : How satisfied are you with your treatment of diabetes?	0 (0.0%)	6 (5.1%)	29 (24.8%)	72 (61.5%)	5 (4.3%)
11^a^: How much can you accept that others know you have diabetes?	4 (3.4%)	12 (10.3%)	21 (17.9%)	40 (34.2%)	39 (33.3%)
12: How much have you adapted to life with diabetes?	3 (2.6%)	5 (4.3%)	27 (23.1%)	59 (50.4%)	23 (19.7%)
13: How often do you feel uncomfortable due to diabetes?	28 (23.9%)	49 (41.9%)	19 (16.2%)	13 (11.1%)	8 (6.8%)
14: How often do you have negative feeling due to diabetes?	46 (39.3%)	33 (28.2%)	12 (10.3%)	16 (13.7%)	10 (8.5%)
15: How satisfied are you with your relationship with your family since you were diagnosed with diabetes?	0 (0.0%)	4 (3.4%)	16 (13.7%)	82 (70.1%)	15 (12.8%)
16: Overall, how satisfied are you with your diabetes care?	0 (0.0%)	1 (0.9%)	7 (6.0%)	83 (70.9%)	26 (22.2%)
17^a^: How much trouble does diabetes cause in your social life?	67 (57.3%)	18 (15.4%)	8 (6.8%)	6 (5.1%)	17 (14.5%)
18^a^: How much trouble does diabetes cause in your leisure life?	60 (51.3%)	24 (20.5%)	7 (6.0%)	10 (8.5%)	14 (12.0%)
19: How much trouble does diabetes cause in your daily activities?	71 (60.7%)	16 (13.7%)	4 (3.4%)	6 (5.1%)	19 (16.2%)
20^a^: How much trouble does diabetes cause in your future expectations?	45 (38.5%)	28 (23.9%)	13 (11.1%)	13 (11.1%)	17 (14.5%)

^a^with missing values; underlined items were retained in the following analyses (as shown in Table 3)

**Table 3:** Item properties in DMQoL

Item #: Item description	Corrected item-total correlation	Factor loading^a^	Infit MnSq^b^	Outfit MnSq^b^
2: How satisfied are you with the time you spend on diabetes care?	0.591	0.677	0.83	0.84
3 How satisfied are you with your expenses on diabetes care?	0.506	0.575	0.88	0.87
4: How satisfied are you with the results of glycemic control?	0.574	0.623	0.94	0.97
5: How satisfied are you with the results of the control of diabetes-related complications?	0.500	0.547	1.09	1.13
7: How satisfied are you with your diet control?	0.583	0.630	0.68	0.74
8: How satisfied are you with your physical activities?	0.573	0.617	1.17	1.13
9: How satisfied are you with your weight control?	0.433	0.470	1.22	1.14
10: How satisfied are you with your treatment of diabetes?	0.526	0.578	0.77	0.81
12: How much have you adapted to life with diabetes?	0.577	0.641	1.43	1.37
15: How satisfied are you with your relationship with your family since you were diagnosed with diabetes?	0.432	0.482	0.89	0.90

^a^Using explorartory factor analysis; ^b^Using Rasch anlaysis

*Infit* information-weighted fit statistic, *Outfit* outlier-sensitive fit statistic, *MnSq* mean square

The internal consistency of the 10 items was satisfactory, as determined using Cronbach’s α (coefficient = 0.84) and Rasch analysis (person separation reliability = 0.81 and item separation reliability = 0.94). The total score of DMQoL was moderately correlated with the score of the two general items on WHOQOL-BREF, with the scores of psychological and environment domains, and with the total score of WHOQOL-BREF. However, low relationships were found between the scores of DMQoL and that of the physical (*r* = 0.177; *p* = 0.058) WHOQOL-BREF domain. After adjusting for age, the correlation between the score of the DMQoL and that of the physical WHOQOL-BREF domain was substantially improved (*r* = 0.283; *p* = 0.003) (Table 4).

**Table 4:** Concurrent validity of DMQoL with World Health Organization Quality of Life-short version (WHOQOL-BREF)

WHOQOL-BREF	*r* (*p*-value)	
	Pearson correlation	Partial correlation^a^
Q1: overall QoL	0.416 (< 0.001)	0.371 (< 0.001)
Q2: general health	0.479 (< 0.001)	0.457 (< 0.001)
Physical domain	0.177 (0.058)	0.283 (0.003)
Psychological domain	0.408 (< 0.001)	0.417 (< 0.001)
Social domain	0.317 (0.001)	0.336 (< 0.001)
Environment domain	0.580 (< 0.001)	0.572 (< 0.001)
Total score	0.461 (< 0.001)	0.481 (< 0.001)

^a^adjusted for age

Finally, known-group validity was supported for DMQoL in laboratory data of HbA1c and cholesterol. Specifically, patients with HbA1c ≤ 7% had significantly higher DMQoL mean scores than did those with HbA1c > 8% (3.66 ± 0.47 vs. 3.41 ± 0.53; *p* = 0.037). In addition, although not significant, our results showed the trend that patients with cholesterol ≤200 mg/dl had higher DMQoL mean scores than did those with cholesterol >200 mg/dl (3.58 ± 0.52 vs. 3.29 ± 0.47; *p* = 0.056). In addition, no significant differences were found in WHOQOL-BREF between patients with HbA1c ≤ 7% and those with HbA1c > 8% (*p* = 0.173 for Q1; 0.228 for Q2; 0.740 for physical domain; 0.345 for psychological domain; 0.134 for social domain; 0.413 for environment domain; 0.411 for total score) and between patients with cholesterol ≤200 mg/dl and those with cholesterol >200 mg/dl (*p* = 0.963 for Q1; 0.695 for Q2; 0.564 for physical domain; 0.528 for psychological domain; 0.647 for social domain; 0.074 for environment domain; 0.970 for total score). In terms of diabetes-related complications, DMQoL did not differentiate patients with complications from those without complications. In addition, significant differences were found between patients with and without cerebral and cardiovascular diseases in the physical domain, psychological domain, and total scores of WHOQOL-BREF (Table 5).

**Table 5:** Known-group validity for DMQoL compared with that of WHOQOL-BREF

		Q1^a^	Q2^a^	Physical^a^	Psychological^a^	Social^a^	Environment^a^	Total score^a^	DMQoL
		Mean ± SD	Mean ± SD	Mean ± SD	Mean ± SD	Mean ± SD	Mean ± SD	Mean ± SD	Mean ± SD
CCD	Yes (*n* = 16)	3.38 ± 0.89	2.81 ± 0.91	10.59 ± 2.11*	11.92 ± 1.93*	11.33 ± 3.11	13.83 ± 1.82	47.69 ± 6.43*	3.50 ± 0.37
	No (*n* = 75)	3.52 ± 0.70	3.01 ± 0.86	12.23 ± 1.67*	13.09 ± 1.89*	12.74 ± 3.44	14.60 ± 1.75	52.68 ± 6.64*	3.46 ± 0.51
PVD	Yes (*n* = 4)	3.50 ± 0.58	3.49 ± 0.75	10.71 ± 2.40	12.67 ± 2.11	11.50 ± 4.51	13.67 ± 2.22	48.55 ± 10.32	3.78 ± 0.40
	No (*n* = 87)	3.25 ± 0.96	2.97 ± 0.87	12.01 ± 1.81	12.90 ± 1.94	12.55 ± 3.38	14.50 ± 1.76	51.99 ± 6.68	3.45 ± 0.49
Retinopathy	Yes (*n* = 16)	3.44 ± 0.73	2.81 ± 0.91	11.86 ± 2.00	13.00 ± 1.46	11.06 ± 5.41	14.86 ± 1.56	50.78 ± 7.44	3.44 ± 0.56
	No (*n* = 75)	3.51 ± 0.74	3.01 ± 0.86	11.98 ± 1.82	12.86 ± 2.03	12.82 ± 2.75	14.38 ± 1.82	52.07 ± 6.73	3.47 ± 0.47
Nephropathy	Yes (*n* = 34)	3.41 ± 0.70	2.82 ± 0.90	11.88 ± 2.02	12.86 ± 2.00	12.44 ± 3.60	14.54 ± 1.70	51.72 ± 7.10	3.52 ± 0.51
	No (*n* = 57)	3.54 ± 0.76	3.07 ± 0.84	12.00 ± 1.74	12.90 ± 1.92	12.55 ± 3.32	14.42 ± 1.83	51.90 ± 6.73	3.43 ± 0.47
CKD stages 3–5, eGFR <60	Yes (*n* = 11)	3.64 ± 0.67	2.55 ± 0.93	12.21 ± 2.17	12.30 ± 2.21	12.27 ± 3.52	14.83 ± 1.77	58.36 ± 4.77	3.61 ± 0.50
	No (*n* = 80)	3.48 ± 0.75	3.04 ± 0.85	11.92 ± 1.80	12.97 ± 1.90	12.54 ± 3.41	14.41 ± 1.78	57.23 ± 7.36	3.45 ± 0.48
Neuropathy	Yes (*n* = 10)	3.50 ± 0.53	3.30 ± 0.82	11.14 ± 1.69	12.27 ± 2.02	10.80 ± 3.33	14.13 ± 2.22	48.34 ± 6.62	3.40 ± 0.68
	No (*n* = 81)	3.49 ± 0.76	2.94 ± 0.87	12.06 ± 1.84	12.96 ± 1.92	12.72 ± 3.38	14.50 ± 1.73	52.28 ± 6.77	3.47 ± 0.46
HbA1c	≤ 7% (*n* = 38)	3.58 ± 0.68	3.26 ± 0.86	11.85 ± 2.40	13.05 ± 2.37	12.49 ± 2.81	14.27 ± 1.94	51.67 ± 7.48	3.66 ± 0.47*
	> 8% (*n* = 32)	3.34 ± 0.75	3.00 ± 0.95	12.46 ± 2.08	13.10 ± 2.16	12.94 ± 3.80	14.83 ± 1.77	53.34 ± 7.77	3.41 ± 0.53*
Cholesterol	≤ 200 mg/dl (*n* = 101)	3.55 ± 0.76	3.06 ± 0.91	12.55 ± 2.33	13.33 ± 2.19	12.86 ± 3.21	14.84 ± 1.77	53.62 ± 7.44	*3.58 ± 0.52* ^#^
	>200 mg/dl (*n* = 13)	3.54 ± 0.88	3.15 ± 0.80	12.57 ± 1.19	12.97 ± 2.17	13.25 ± 3.70	13.91 ± 1.86	52.77 ± 8.14	*3.29 ± 0.47* ^#^

*p < 0.05; ^#^ *p* < 0.06 *CCD* cerebral and cardiovascular diseases, *PVD* peripheral vascular diseases, *CKD* chronic kidney diseases; ^a^From WHOQOL-BREF

## Discussion

With a growing population suffering from diabetes and its associated serious short- and long-term consequences, it is important to assess how this chronic disease influences patients’ perceived health (i.e., HRQoL) in addition to laboratory or diagnostic tests. With this information, holistic care and effective treatment can be provided to optimize the HRQoL of patients. This study developed a brief version of an HRQoL instrument with only 10 items in the final scale, called DMQoL, specific to patients with diabetes. DMQoL can be used stand-alone in daily practice or in conjunction with the WHOQOL-BREF for measuring the overall HRQoL of a patient. The DMQoL content was constructed based on a rigorous methodology that included extensive literature review, panel discussion, and patient interviews. The item properties of the10 items in the final scale were supported; the preliminary results of their reliability and validity were satisfactory. DMQoL has a high level of internal consistency, as shown by the Cronbach’s α coefficient and separation reliabilities in Rasch models. The corrected item-total correlation for all items was above 0.4, implying that the items correlated well with the scale overall. The concurrent validity of DMQoL was confirmed by significantly moderate relationships between the score of DMQoL and those of an existing validated HRQoL measure (i.e., WHOQOL-BREF). The significant difference in the scores of DMQoL between patients with different levels of glycemic control (i.e., HbA1c ≤ 7%, > 8%) is evidence of its known-group validity.

Previous research demonstrated an inverse association between HbA1c and HRQoL in patients with diabetes. Studies from the United States showed that improved HbA1c is associated with better HRQoL [32], fewer patient-reported diabetes symptoms, lower symptom distress, and higher treatment satisfaction [33]. Glycaemic change based on HbA1c was associated with diabetes-specific quality of life (measured using Audit of Diabetes-Dependent Quality of Life; ADDQoL [34], Diabetes Health Profile [16]), but not with generic health status (measured using SF-36 [33, 35], RAND-36 [14, 16]). These results imply that diabetes-specific HRQoL measurements are more sensitive and responsive to glycemic changes or differences than is a generic instrument. Consistently, we found that DMQoL as a diabetes-specific HRQoL instrument is responsive to subgroup differences in HbA1c, whereas WHOQOL-BREF, a generic HRQoL instrument, is not. This result supports that DMQoL is a discriminative instrument for clinical differences such as HbA1c in patients with diabetes. There is growing support for the long-term health benefits of tight blood glycemic control early in the diabetes disease trajectory [36]. Hence, it is important to apply diabetes-specific HRQoL for those at early stages of diabetes when the burden of glycemic control and treatment may be higher than the burden of the disease and its long-term complications, and to early detect the effects of glycemic changes on the HRQoL of patients for preventing long-term risks of diabetes-related complications.

Several studies have shown significantly negative impacts of diabetes-related complications on patients’ HRQoL measured by using generic measures such as WHOQOL-BREF [37, 38]; in contrast, we found no significant difference in DMQoL scores between patients with and without diabetes-related complications. One possible explanation for our finding is that our participants were less severe cases with few diabetes-related complications (Table 1). So, the number of diabetes-related complication events/cases was limited in our analyses, which comprised the power for the statistical testing. Moreover, during face-to-face interviews, we found that most participants less often recognized diabetes-related complications and their impacts on daily life. As compared to the signs and symptoms of high or low blood glucose, long-term or chronic diabetes-related complications that develop over many years may not be easy to recognize (e.g., how these complications are associated with diabetes itself, how poor glycemic control is linked to chronic or long-term diabetes-related complications). Since many chronic complications produce no symptoms in the early stages, effective education and communication are needed to help patients understand chronic diabetes-related complications and their consequents for their health and quality of life. However, our findings (Table 5) are consistent with previous studies, showing that WHOQOL-BREF is discriminative of subgroup differences in diabetes-related complications such as macrovascular diseases [37] and that the presence of complications is the most powerful variable influencing the physical domain of HRQoL [38]. This implies that for those with severe/advanced cases suffering from diabetes-related complications, the combination of DMQoL and WHOQOL-BREF is recommended which will enhance the instrument’s ability to detect clinical meaningful changes in HRQoL of diabetes.

There are several advantages of DMQoL. First, as compared to generic HRQoL measures (e.g., WHOQQL-BREF), DMQoL is more sensitive to glycemic difference, as shown by the results of known-group validity. The significant difference in the scores of DMQoL between individuals with different HbA1c levels supports the ability of DMQoL to differentiate patients with diabetes in poor or well glycemic control. Therefore, its score may help healthcare providers to detect the impact of diabetes on their patients in the early stage of diabetes progression (i.e., based on HbA1c changes). In contrast, WHOQOL-BREF may not be as effective (sensitive) as DMQoL in detecting early changes in the HRQoL of patients with diabetes. For advanced cases with diabetes-related complications, a combination of DMQoL and WHOQOL-BREF is preferred. Second, compared to existing diabetes-specific HRQoL instruments (Additional file 1: Appendix 1), DMQoL is a much shorter measure with only 10 items. In particularly, existing diabetes-specific HRQoL instruments in Chinese, which have more than 39 items [13–15], may be too long for practical purposes such as clinical evaluation. With only 10 items, DMQoL can be completed within 5 min, and is easily administered in routine practice. Also, it can be used as a screening tool to capture a cross-sectional snapshot of patient outcomes, especially in large-scale audits of practice outcomes. Physicians could follow these outcomes longitudinally as a way to compare care against normative values and to identify areas for clinical attention or improvement. Also, a simplified HRQoL measure such as DMQoL is an attractive research instrument for population studies because it is more time-efficient, reduces responder burden, and thus can minimize missing data. The length of a questionnaire clearly influences response rates and quality of data [39, 40]. Long scales increase responder burden and the cost of survey production, distribution, and coding [39, 40]. The brief DMQoL thus offers greater efficiency over existing diabetes mellitus measures by reducing responder burden and eliminating the need for complicated scoring algorithms. Third, DMQoL can be used as an adjunct to WHOQOL-BREF when clinicians or researchers want to understand the overall HRQoL of patients and to compare HRQoL across groups with different types of conditions/illnesses. Hence, the combination of the WHOQOL-BREF and its supplemental module, DMQoL, will enhance the ability of the instrument to detect clinical meaningful changes associated with diabetes progressions and its treatments.

The results of this study should be interpreted with caution due to several limitations. First, our study population was predominately patients with type 2 diabetes, middle to old age, and relatively healthy (i.e., few diabetes-related complications). Therefore, the generalizability of our results for some diabetes-related complications needs to be done cautiously. Future studies on patients with type 1 diabetes and other age groups or severe diabetes cases are warranted to validate DMQoL. Second, test-retest reliability and the responsiveness of DMQoL have not been confirmed yet, highlighting the need for future research. Specifically, healthcare providers may want to know whether changes in scores reflect meaningful changes in HRQoL. Third, although we proposed using only 10 items for DMQoL, the item properties were examined using 20 items. Therefore, we cannot ensure that the item properties were unchanged for the 10 items in DMQoL. Although we believe that the psychometric properties will be improved if a shorter version (i.e., using a 10-item version instead of a 20-item version) is used, future studies are needed to corroborate our postulation. Fourth, we did not collect the data of other existing diabetes-specific HRQoL instruments. Therefore, we were unable to test the construct validity between DMQoL and other diabetes-specific HRQoL instruments. Lastly, since there is no defined optimal number of items in the scale, further refinement and simplification of DMQoL are recommended to facilitate its administration in clinical practice and studies.

## Conclusion

Based on our psychometric findings, the DMQoL can serve as an efficient screening tool in routine practice for patients with diabetes and as a research instrument with relatively low administration burden and cost of production. It can be used stand-alone for quickly screening the impact of diabetes progression in early phases (e.g., glycemic changes) on sufferers or combined with WHOQOL-BREF for assessing the overall HRQoL of patients. Future research is needed to validate DMQoL across diabetes patients with different ages, complications, comorbidity, and severity and in different clinical settings and cultures.

## Additional file

Additional file 1: Appendix 1. Existing diabetes-specific health-related quality life measures. **Appendix 2.** Initial 20 items in DM module in Chinese (Bold items: 10 items retained in the final scale). **Appendix 3.** Initial 20 items in DM module in English (Bold items: 10 items retained in the final scale). (DOCX 30 kb)

## Abbreviations

ADDQoL: Audit of diabetes-dependent quality of life
CVD: Cardiovascular diseases
DIMS: Diabetes impact management scales
DMQoL: Diabetes-specific quality of life questionnaire module
DQOL: Diabetes quality of life
EFA: Exploratory factor analysis
e-GFR: estimated glomerular filtration rate
HRQoL: Health-related quality of life
NCKUH: National Cheng Kung University Hospital
PA: Parallel analysis
WHOQOL-BREF: World Health Organization Quality of Life scale brief version
