# Supplementary material for: Development of diabetes-specific quality of life module to be in conjunction with the World Health Organization quality of life scale brief version (WHOQOL-BREF)
Source: Health Qual Life Outcomes. 2017 Aug 23;15:167. doi: 10.1186/s12955-017-0744-3 (PMC5569515; doi:10.1186/s12955-017-0744-3)
Supplement: Additional file 1: Appendix 1. — Existing diabetes-specific health-related quality life measures. Appendix 2. Initial 20 items in DM module in Chinese (Bold items: 10 items retained in the final scale). Appendix 3. Initial 20 items in DM module in English (Bold items: 10 items retained in the final scale). (DOCX 30 kb) [file 12955_2017_744_MOESM1_ESM.docx]

**Appendix 1: Existing diabetes-specific health-related quality life measures**

1. Carey, M. P., Jorgensen, R. S., Weinstock, R. S., Sprafkin, R. P., Lantinga, L. J., Carnrike Jr, C., Baker, M. T., & Meisler, A. W. (1991). Reliability and validity of the appraisal of diabetes scale. Journal of behavioral medicine, 14(1), 43-50.
2. Bradley, C., Todd, C., Gorton, T., Symonds, E., Martin, A., & Plowright, R. (1999). The development of an individualized questionnaire measure of perceived impact of diabetes on quality of life: the ADDQoL. Quality of Life Research, 8(1-2), 79-91.
3. Boyer, J. G., & Earp, J. A. L. (1997). The development of an instrument for assessing the quality of life of people with diabetes: Diabetes-39. Medical care, 35(5), 440-453.
4. Fitzgerald, J. T., Davis, W. K., Connell, C. M., Hess, G. E., Funnell, M. M., & Hiss, R. G. (1996). Development and validation of the Diabetes Care Profile. Evaluation & the health professions, 19(2), 208-230.
5. Polonsky, W. H., Fisher, L., Earles, J., Dudl, R. J., Lees, J., Mullan, J., & Jackson, R. A. (2005). Assessing psychosocial distress in diabetes development of the diabetes distress scale. Diabetes care, 28(3), 626-631.
6. Meadows, K., Steen, N., McColl, E., Eccles, M., Shiels, C., Hewison, J., & Hutchinson, A. (1996). The Diabetes Health Profile (DHP): a new instrument for assessing the psychosocial profile of insulin requiring patients—development and psychometric evaluation. Quality of Life Research, 5(2), 242-254.
7. Hammond, G. S., & Aoki, T. T. (1992). Measurement of health status in diabetic patients: diabetes impact measurement scales. Diabetes Care, 15(4), 469-477.
8. Shen, W., Kotsanos, J. G., Huster, W. J., Mathias, S. D., Andrejasich, C. M., & Patrick, D. L. (1999). Development and validation of the diabetes quality of life clinical trial questionnaire. Medical care, AS45-AS66.
9. Group, D. R. (1988). Reliability and validity of a diabetes quality-of-life measure for the diabetes control and complications trial (DCCT). Diabetes care, 11(9), 725-732.
10. Bott, U., Mühlhauser, I., Overmann, H., & Berger, M. (1998). Validation of a diabetes-specific quality-of-life scale for patients with type 1 diabetes. Diabetes care, 21(5), 757-769.
11. Araki, A., & Ito, H. (2003). Development of elderly diabetes burden scale for elderly patients with diabetes mellitus. Geriatrics & Gerontology International, 3(4), 212-224.
12. Peyrot, M., & Rubin, R. R. (2005). Validity and Reliability of an Instrument for Assessing Health-Related Quality of Life and Treatment Preferences The Insulin Delivery System Rating Questionnaire. Diabetes Care, 28(1), 53-58.
13. Snoek, F. J., Pouwer, F., Welch, G. W., & Polonsky, W. H. (2000). Diabetes-related emotional distress in Dutch and US diabetic patients: cross-cultural validity of the problem areas in diabetes scale. Diabetes care, 23(9), 1305-1309.

**Appendix 2: Initial 20 items in DM module in Chinese (Bold items: 10 items retained in the final scale)**

|  |
| --- |
| 1. 整體來說，您覺得糖尿病控制對您的生活有影響嗎？ |
| □完全沒有 □有一點有 □中等程度有 □很有 □極有 |
| **2. 您滿意自己花在糖尿病照顧上的時間嗎？** |
| **□極不滿意 □不滿意 □中等程度滿意 □滿意 □極滿意** |
| **3. 您滿意自己花在糖尿病照顧上的金錢費用嗎？** |
| **□極不滿意 □不滿意 □中等程度滿意 □滿意 □極滿意** |
| **4. 您滿意自己血糖控制的結果嗎？** |
| **□極不滿意 □不滿意 □中等程度滿意 □滿意 □極滿意** |
| **5.您滿意自己糖尿病相關併發症(例如:腎病變、眼睛病變、神經病變、冠狀動脈疾病、中風等等)的控制結果嗎？** |
| **□極不滿意 □不滿意 □中等程度滿意 □滿意 □極滿意** |
| 6. 您自己要做到糖尿病的自我照護會有困難嗎？(例如: 測血糖、飲食控制、運動、用藥) |
| □完全沒有困難 □有一點困難 □中等程度困難 □很困難 □極困難 |
| **7. 您滿意自己的飲食型態調整嗎？** |
| **□極不滿意 □不滿意 □中等程度滿意 □滿意 □極滿意** |
| **8. 您滿意自己的運動習慣嗎？** |
| **□極不滿意 □不滿意 □中等程度滿意 □滿意 □極滿意** |
| **9. 您滿意自己的體重控制嗎？** |
| **□極不滿意 □不滿意 □中等程度滿意 □滿意 □極滿意** |
| **10. 您滿意自己的糖尿病藥物治療嗎？** |
| **□極不滿意 □不滿意 □中等程度滿意 □滿意 □極滿意** |
| 11. 您能接受讓其他人知道您有糖尿病嗎？ |
| □完全不能夠 □少許能夠 □中等程度能夠 □很能夠 □完全能夠 |
| **12. 您能適應自己有糖尿病(的生活)嗎？** |
| **□完全不能夠 □少許能夠 □中等程度能夠 □很能夠 □完全能夠** |
| 13. 糖尿病會讓您感到身體不適嗎(例如:無力、頭暈、飢餓、發抖、冒冷汗等症狀)？ |
| □從來沒有 □不常有 □一半有一半沒有 □很常有 □一直都有 |
| 14. 您會因為自己有糖尿病而有負面感受嗎？(例如: 傷心、緊張、焦慮、憂鬱) |
| □從來沒有 □不常有 □一半有一半沒有 □很常有 □一直都有 |
| **15. 您自從有糖尿病以後，您滿意您與家人的關係嗎？** |
| **□極不滿意 □不滿意 □中等程度滿意 □滿意 □極滿意** |
| 16. 整體來說，您滿意您目前所接受的糖尿病照護嗎？(例如: 醫師、護理師、營養師、藥師等) |
| □極不滿意 □不滿意 □中等程度滿意 □滿意 □極滿意 |
| 17. 糖尿病對您的社交生活有造成困擾嗎？ |
| □完全沒有 □有一點有 □中等程度有 □很有 □極有 |
| 18. 糖尿病對您的休閒生活有造成困擾嗎？ |
| □完全沒有 □有一點有 □中等程度有 □很有 □極有 |
| 19. 糖尿病對您的日常生活的活動有造成困擾嗎？(例如: 職場工作, 家事) |
| □完全沒有 □有一點有 □中等程度有 □很有 □極有 |
| 20. 糖尿病對您的未來期待有造成困擾嗎？(例如: 生活、收入、健康) |
| □完全沒有 □有一點有 □中等程度有 □很有 □極有 |

**Appendix 3: Initial 20 items in DM module in English (Bold items: 10 items retained in the final scale)**

1. Overall, how much does diabetes control influence your life?

□ None at all □ Slightly □ Moderately □ Very □ Extremely

1. **How satisfied are you with the time you spend on diabetes care?**

**□ Very dissatisfied □ Dissatisfied □ Neither satisfied nor dissatisfied □ Satisfied □ Very satisfied**

1. **How satisfied are you with your expenses on diabetes care?**

**□ Very dissatisfied □ Dissatisfied □ Neither satisfied nor dissatisfied □ Satisfied □ Very satisfied**

1. **How satisfied are you with the results of glycemic control?**

**□ Very dissatisfied □ Dissatisfied □ Neither satisfied nor dissatisfied □ Satisfied □ Very satisfied**

1. **How satisfied are you with the results of the control of diabetes-related complications (e.g., nephropathy, retinopathy, neuropathy, coronary artery disease, and stroke)?**

**□ Very dissatisfied □ Dissatisfied □ Neither satisfied nor dissatisfied □ Satisfied □ Very satisfied**

1. How much difficulty have you had in self-care in the management of diabetes (e.g., checking blood sugar, diet, exercise, or taking diabetes pills)?

□ None at all □ Slightly □ Moderately □ Very □ Extremely

1. **How satisfied are you with your diet control?**

**□ Very dissatisfied □ Dissatisfied □ Neither satisfied nor dissatisfied □ Satisfied □ Very satisfied**

1. **How satisfied are you with your physical activities?**

**□ Very dissatisfied □ Dissatisfied □ Neither satisfied nor dissatisfied □ Satisfied □ Very satisfied**

1. **How satisfied are you with your weight control?**

**□ Very dissatisfied □ Dissatisfied □ Neither satisfied nor dissatisfied □ Satisfied □ Very satisfied**

1. **How satisfied are you with your treatment of diabetes?**

**□ Very dissatisfied □ Dissatisfied □ Neither satisfied nor dissatisfied □ Satisfied □ Very satisfied**

1. How much can you accept that others know you have diabetes?

□ None at all □ A little □ A moderate amount □ Very much □ An extremely amount

1. **How much have you adapted to life with diabetes?**

**□ None at all □ Slightly □ Moderately □ Very □ Extremely**

1. How often do you feel uncomfortable due to diabetes? (e.g., weakness, dizziness, hunger, shivering, sweating, or other symptoms)

□ Never □ Seldom □ Quite often □ Very often □ Always

1. How often do you have negative feeling due to diabetes? (e.g., sadness, stress, anxiety, or depression)

□ Never □ Seldom □ Quite often □ Very often □ Always

1. **How satisfied are you with your relationship with your family since you were diagnosed with diabetes?**

**□ Very dissatisfied □ Dissatisfied □ Neither satisfied nor dissatisfied □ Satisfied □ Very satisfied**

1. Overall, how satisfied are you with your diabetes care? (i.e., physician, nurse, dietitian, and pharmacist)

**□ Very dissatisfied □ Dissatisfied □ Neither satisfied nor dissatisfied □ Satisfied □ Very satisfied**

1. How much trouble does diabetes cause in your social life?

□None at all □A little □A moderate amount □Very much □An extremely amount

1. How much trouble does diabetes cause in your leisure life?

□ None at all □A little □A moderate amount □ Very much □An extremely amount

1. How much trouble does diabetes cause in your daily activities (e.g., job, chores)?

□ None at all □A little □A moderate amount □ Very much □An extremely amount

1. How much trouble does diabetes cause in your future expectations (e.g., life, income, health)?

□ None at all □A little □A moderate amount □ Very much □An extremely amount
